# Treatment outcomes of fixed-dose combination versus separate tablet regimens in pulmonary tuberculosis patients with or without diabetes in Qatar

**DOI:** 10.1186/s12879-017-2231-1

**Published:** 2017-02-02

**Authors:** Mohammad H. Al-Shaer, Hanine Mansour, Hazem Elewa, Pascale Salameh, Fatima Iqbal

**Affiliations:** 1 Department of Pharmacy, Al Wakra Hospital – Hamad Medical Corporation, Doha, Qatar; 2 Present address: Department of Pharmacotherapy and Translational Research, Infectious Disease Pharmacokinetics Laboratory, College of Pharmacy and Emerging Pathogens Institute, University of Florida, Gainesville, FL 32610 USA; 3 School of Pharmacy, Lebanese American University, P.O Box 36-S 23, Byblos, Lebanon; 4 College of Pharmacy, Qatar University, Doha, Qatar; 5 Present address: Faculty of Medical Sciences, Lebanese University, Beirut, Lebanon; 6 Department of Pharmacy, Rumailah Hospital – Hamad Medical Corporation, Doha, Qatar

**Keywords:** Pulmonary tuberculosis, Fixed-dose, Separate tablets, Effectiveness, Safety

## Abstract

**Background:**

Tuberculosis is considered the second most common cause of death due to infectious agent. The currently preferred regimen for treatment of pulmonary tuberculosis (PTB) is isoniazid, rifampin, pyrazinamide, and ethambutol, which has been used either as separate tablets (ST) or as fixed-dose combination (FDC). To date, no studies have compared both regimens in Qatar. We aim to evaluate the safety and effectiveness of FDC and ST regimen for treating PTB, in addition to comparing safety and efficacy of FDC and ST regimens in patients with diabetes treated for TB.

**Methods:**

A retrospective observational study was conducted in two general hospitals in Qatar. Patients diagnosed with PTB received anti-tuberculosis medications (either as FDC or ST) administered by the nurse. Sputum smears were tested weekly. We assessed the time to negative sputum smear and incidence of adverse events among FDC and ST groups.

**Results:**

The study included 148 patients. FDC was used in 90 patients (61%). Effectiveness was not different between FDC and ST regimens as shown by mean time to sputum conversion (29.9 ± 18.3 vs. 35.6 ± 23 days, *p* = 0.12). Similarly, there was no difference in the incidence of adverse events, except for visual one that was higher in ST group. Among the 33 diabetic patients, 19 received the FDC and had faster sputum conversion compared to those who received ST (31 ± 12 vs. 49.4 ± 30.9 days, *p* = 0.05). Overall, diabetic patients needed longer time for sputum conversion and had more hepatotoxic and gastric adverse events compared to non-diabetics.

**Conclusion:**

ST group had higher visual side effects compared to FDC. FDC may be more effective in diabetic patients; however, further studies are required to confirm such finding.

## Background

Tuberculosis (TB) is the second greatest worldwide infectious killer. According to the World Health Organization (WHO), 37 million lives were treated between 2000 and 2013. In 2014, 9.6 million people fell ill with TB [1]. Qatar is a peninsula located between the Persian Gulf and Saudi Arabia [2]. In January 2016, the population was estimated to be over 2.3 million compared to around 220 thousands in the mid-1980s. This increase was primarily driven by the expatriate workforce traveling to Qatar. In 2013, number of foreign labors constituted around 85.7% of the population and up to 94.1% of the country’s workforce [3]. The majority of this workforce is from Asian countries with high TB prevalence such as India (31%), Nepal (23%), Bangladesh (4.8%) leading to an increase in the total number of TB [3]. In Qatar, all cases of TB are registered at the Communicable Disease Control and Prevention Section in the Supreme Council of Health [4]. TB cases have increased from 184 cases in 1990 to 728 cases in 2012 with a decrease in the incidence rate from 44/100,000 to 41/100,000. A decrease in the incidence of TB was recorded at 40/100,000 with 511 new cases [5]. *Mycobacterium tuberculosis* is the bacteria responsible for TB and it spreads through the air leading to lung infection which is the most common TB infection. Even though TB is preventable and curable, achieving a high cure rate is challenging and critical. The usual TB treatment regimen is a standard 2-month regimen of isoniazid (INH), rifampin (RIF), pyrazinamide (PZA), and ethambutol (EMB), followed by a 4-month regimen of isoniazid and rifampin. This classic regimen is associated with poor compliance and adherence which leads to the development of resistant strains and multidrug resistant bacilli [6]. Several methods have been explored to improve compliance and reduce errors such as the fixed-dose combination (FDC). The rationale of FDC is that the presence of all these drugs combined in one tablet can facilitate dosage calculation, prevent prescribing errors, increases patient’s acceptance, and decreases pill burden [7–9]. During the 1980s and 1990s, health care providers were concerned about the quality of FDCs where poor bioavailability of the rifampin component of the tablet was an issue [10, 11]. However, current FDCs are fully bioequivalent, stable and efficacious even after 6 months in tropical conditions [7, 12–17]. Common fixed-dose combinations (FDCs) are of two anti-tuberculosis drugs (2FDCs, usually [RIF] + [INH]), three drugs (3FDCs, RIF + INH + [PZA]) and four drugs (4FDCs, RIF + INH + PZA + [EMB]). Several studies tested the safety and efficacy in different parts of the world using different brands of FDC regimens. However, no similar studies were performed in Qatar.

The objective of this study is to compare the FDC using Rifafour® for the first time as part of an FDC regimen and compare it to the regimens of separate tablets (ST) during the initial phase of TB treatment in Qatar, while assessing the safety and effectiveness of both regimens in terms of adverse events and time to negative sputum smear, respectively. The secondary objective is to compare FDC to ST regimens in terms of safety and efficacy in patients with diabetes treated for TB.

## Methods

### Study settings

This study was performed in two general hospitals in Qatar, with a total of 450 beds. Pulmonary tuberculosis (PTB) patients were diagnosed based on their symptoms, chest X-rays, and sputum smears. Once diagnosis was confirmed by positive sputum smear, patients were kept in isolation rooms within the hospital until their sputum smears became negative, confirmed by two consecutive smears. Medical teams started patients on first-line anti-TB medications (RIF, INH, PZA, and EMB), either as FDC (Rifafour® [Sanofi-Aventis]: RIF 150, INH 75, PZA 400, and EMB 275 mg/tablet) or ST (RIF 150, INH 100 or 300, PZA 500, and EMB 400 or 500 mg/tablet), administered by the nurse early morning on empty stomach. The dose was calculated based on the patient’s weight. Table 1 shows the daily doses by body weight category for each regimen. Choice of the regimen was based on the introduction of the FDC in June 2013, at that time physicians started prescribing the FDC for most patients. Patients received pyridoxine 40 mg orally once daily along with anti-TB medications. Diabetes was controlled during the hospital stay with oral hypoglycemic agents and/or insulin, along with regular random blood sugar monitoring. Also, hypertension was controlled by oral antihypertensive agents. Those who had vitamin D deficiency were started on vitamin D oral supplements in the hospital. Sputum samples were sent regularly to the microbiology laboratory on a weekly basis for AFB testing, and the highest bacillary load was recorded based on WHO/International Union Against Tuberculosis and Lung Disease (IUATLD) system [18]. This study was approved by Hamad Medical Corporation institutional review board. All procedures performed in this study were in accordance with the 1964 Helsinki declaration. For this type of study formal consent is not required.

**Table 1:** Daily doses by body weight category for FDC and ST groups

Weight, kg	FDC, (# of tablet^a^)	FDC, mg				ST, mg			
		INH	RIF	PZA	EMB	INH	RIF	PZA	EMB
<35	2	150	300	800	550	150	300	750	800
35–54	3	225	450	1200	825	225	450	1000	1200
>54	4	300	600	1600	1100	300	600	1500	1600

*EMB* ethambutol, *FDC* fixed-dose combination, *INH* isoniazid, *PZA* pyrazinamide, *RIF* rifampin, *ST* separate tablets

^a^FDC tablet contains rifampin 150 mg, isoniazid 75 mg, pyrazinamide 400 mg, and ethambutol 275 mg

### Study design

In this retrospective cohort study, we compared FDC and ST in terms of effectiveness and safety. We included all patients diagnosed with PTB based on the positive sputum AFB results, admitted to the hospital between December 2012 and November 2014, and 18 years old or more. Patients with mycobacteria resistant to the first-line anti-TB were excluded.

### Data collection

We identified patients from the hospital admission database, while medical records and laboratory and pharmacy databases were used to collect patients’ demographics, comorbidities (e.g. hypertension and diabetes), vitamin D levels, anti-TB regimens and adverse events (cutaneous, gastric, musculoskeletal, hepatic, visual, and hyperuricemia), and time to negative sputum AFB.

Hepatotoxicity was defined as elevation in transaminases 3 times or more above upper normal limit (>120 units/L). Cutaneous adverse events were defined as rash or itching, and were identified from physician notes and antihistamine administration once reported by the patient. Gastric adverse events including nausea, vomiting, or epigastric and abdominal pain were identified. Hyperuricemia was defined as any uric acid level above 416 micromol/L. Visual adverse events including blurred vision, color blindness, or eye pain were recorded when reported by the patient, in addition to the musculoskeletal adverse events such as muscular and joint pain. The primary outcome was to assess the effectiveness of both regimens by assessing time to negative sputum AFB confirmed by two consecutive smears. Secondary outcomes were to assess the safety of regimens by comparing incidence of adverse events between regimens and to compare the effectiveness and safety of both regimens in the different subgroups.

### Statistical analysis

Continuous data were reported as means and standard deviation (SD), whereas categorical data were reported as frequency and percentages. We used student *t*-test to compare continuous data, and Chi-square test to compare categorical data between the two groups of comparison. We set the significance level of 0.05. All statistical analyses were performed using SPSS 19.0 (SPSS Inc. Chicago, IL).

## Results

A total of 148 patients were included in this study (Table 2). The mean (±SD) for age and body mass index (BMI) were 33.9 ± 10.1 years and 20.4 ± 3.1 kg/m^2^, respectively. Patients were mostly of male gender (85.8%) and of Asian ethnicity (87%). A total of 33 patients had diabetes, and 7 patients had hypertension on admission. Nine patients (6%) had vitamin D level <10 ng/ml, and 94 patients (64%) between 10–29 ng/ml. There were 90 patients (61%) and 58 patients (39%) in FDC and ST groups, respectively. Asians accounted for 88.9% in the FDC group and 84.5% in the ST group. The numbers of smokers and male patients were higher in the FDC group than the ST group (44.4% vs. 19%, *p* = 0.001) and (92.2% vs. 75.9%, *p* = 0.005), respectively. There were no other differences between groups in all other baseline characteristics.

**Table 2:** Baseline characteristics and doses of anti-tuberculosis medications

	FDC (*n* = 90)	ST (*n* = 58)
Age in years, mean ± SD	34.3 ± 10.1	33.4 ± 10.2
Male gender, n (%)	83 (92.2)	44 (75.9)
Asian nationality^a^, n (%)	80 (88.9)	49 (84.5)
BMI (kg/m^2^), mean ± SD^b^	20.3 ± 3.1	20.5 ± 3.2
Diabetes, n (%)	19 (21.1)	14 (24.1)
Hypertension, n (%)	4 (4.4)	3 (5.2)
Smoking, n (%)	40 (44.4)	11 (19)
Vitamin D level (ng/ml), mean ± SD^c^	19.7 ± 10	18.5 ± 6.2
Isoniazid (mg/kg), mean ± SD	4.8 ± 0.4	5.5 ± 1
Rifampin (mg/kg), mean ± SD	9.6 ± 0.9	10 ± 1.2
Pyrazinamide (mg/kg), mean ± SD	25.8 ± 2.2	23.4 ± 2.3
Ethambutol (mg/kg), mean ± SD	17.7 ± 1.7	17 ± 5.7
+++ AFB load, n (%)	34 (37.8)	24 (41.4)

*AFB* acid-fast bacilli, *BMI* body mass index, *FDC* fixed-dose combination, *ST* separate tablets

^a^South Asian and Southeast Asian nationalities including Indian, Nepalese, Pakistani, Bangladeshi, Sri Lankan, Indonesian, and Filipino

^b^Data available for 81 (FDC group) and 55 (ST group) patients

^c^Data available for 68 (FDC group) and 49 (ST group) patients

### Effectiveness

There was no statistical difference in the mean time to achieve negative sputum AFB in the FDC and ST regimens groups (Table 3). Even though the objective of this study was not to compare the response to treatment in patients with diabetes; it is worth noting that among the 33 patients with diabetes, 19 (57.6%) received the FDC while the remaining 14 patients (42.4%) received ST. Patients with Diabetes receiving FDC had faster conversion to negative sputum smear compared to those receiving ST (31 ± 12 vs. 49.4 ± 30.9 days, *p* = 0.05). Also, when we compared diabetic patients with different AFB load, only those who received FDC and had AFB +++ (*n* = 8) showed significant faster sputum conversion compared to those who received ST (*n* = 6) (37.8 ± 14.2 vs. 72.5 ± 27.7 days, *p* = 0.01). When compared to non-diabetics, patients with diabetes needed longer time for sputum AFB conversion (38.8 vs. 30.2 days, *p* = 0.03). Time to negative smear was not significantly different between FDC and ST in any of the other subgroups.

**Table 3:** Mean time to negative sputum smear among groups in days

Patients groups	FDC, mean ± SD (n)	ST, mean ± SD (n)	*p*-value^a^
All patients	29.9 ± 18.3 (90)	35.6 ± 23 (58)	0.12
Diabetic	31 ± 12 (19)	49.4 ± 30.9 (14)	0.05
Non-diabetics	29.6 ± 19.7 (71)	31.2 ± 18.2 (44)	0.67
Smokers	26.6 ± 11.3 (40)	35.8 ± 22.9 (11)	0.22

*FDC* fixed-dose combination, *ST* separate tablets, *NS* non-significant

^a^Using independent *t*-test

### Safety

**Fig. 1:**
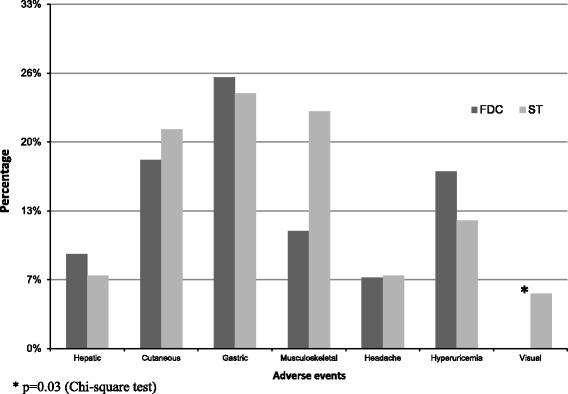
FDC, fixed-dose combination; ST, separate tablets. * *p* = 0.03 (Chi-square test)

The incidence of adverse events between both groups is shown in Fig. 1. None showed statistically significant difference except for visual adverse events that were higher in ST group (5.2% vs. 0%, *p* = 0.03). There was a tendency towards increase in musculoskeletal adverse events in ST group (22% vs. 11%, *p* = 0.06). Subgroup analysis of adverse events between groups showed that in non-diabetics, ST group had higher incidence of musculoskeletal adverse events (25% vs. 9.9%, *p* = 0.03), while other adverse events showed no difference. Diabetic patients had more hepatotoxic and gastrointestinal adverse events compared to non-diabetics (18.2% vs. 5.2%, *p* = 0.016) and (54.5% vs. 16.5% *p* < 0.001), respectively.

## Discussion

This is the first study to compare the safety and efficacy of the four-drug FDC (Rifafour) and ST regimens on the population living in Qatar. Both regimens showed similar efficacy and safety profiles. The efficacy was observed by time to negative sputum AFB during hospitalization and the safety was reported in terms of commonly seen adverse events for TB medications. Such results are consistent with what has been reported in previous trials with few exceptions. For instance, time to negative AFB was tested in the outpatient setting at 2, 4 and 6 months whereas in the current study, it was assessed upon diagnosis and weekly. Also, safety parameters such as musculoskeletal and visual disturbances were observed more in the ST group compared to the FDC group, noting a statistically significance difference in terms of visual disturbances [19, 20]. Bartacek and colleagues studied Rimstar®, a 4-FDC drug, where they compared it to ST regimens in terms of efficacy, safety and patients’ acceptability in an open multicenter, multinational study. Sputum smear conversion was tested at 2, 4, and 6 months then at follow up appointments at 9 and 12 months. The two groups showed similar efficacy, and non-inferiority of 4-FDC to ST was proved. In terms of safety, 4-FDC group showed to have less gastrointestinal disturbance such as nausea and vomiting (*P* = 0.03), liver and biliary disorders such as hepatitis and jaundice (*p* = 0.04), and general disorders such as headache and asthenia (*P* = 0.01) than ST group. Acceptability and convenience was proven to be better with the FDC group than the ST group [19]. In the study C randomized controlled trial, Lienhardt and colleagues evaluated the safety and efficacy of 4- FDC regimen compared to ST in TB patients. Cultures were taken at 2 months and 18 months after the initiation of treatment. FDC showed non-inferiority to ST regimen in terms of efficacy. Safety was measured by looking at adverse events in both groups for the first 2 months of treatment. In terms of safety parameters such as dermatologic, rheumatologic, hepatic, or gastrointestinal disorders, there was no significant difference between the FDC and ST group (*p* = 0.1) [21]. Wu and colleagues conducted a prospective, open label randomized trial comparing FDC and ST regimens in terms of safety and efficacy. Direct observed therapy (DOT) was adapted 5 days a week, while doses were self-administered on weekends. The FDC group received Rifater® (Gruppo Lepetit SPA, Lainate, Italy) which contains (INH/RIF/PZA: 80/120/250 mg per tablet) and EMB for the first two months, followed by Rifinahs® (Gruppo Lepetit SPA, Lainate, Italy) which contains (INH/RIF 100/150 mg per tablet) and EMB for an additional four months or longer. In this study the FDC was a 3 FDC in addition to ethambutol as a separate drug. Efficacy was assessed by examining the sputum culture at 2 and 4 months and then reexamined at 6 months and one year. Both groups showed similar efficacy. In terms of safety, FDC groups showed a transient increase in bilirubin level during treatment compared to ST group [20]. Systemic reviews comparing fixed-dose combination therapy to separate drug combinations were conducted. These reviews had similar conclusions indicating no advantage in terms of efficacy between the two regimens for patients with active TB [22, 23].

In terms of safety, as noted in the results visual adverse events were found higher in the ST group than the FDC group. This could be related to the EMB dose used in the ST regimen compared to the FDC regimen where patients in the ST regimen received a weight based dose estimated around 20 mg/kg whereas in the FDC group received a 15 mg/kg dose. Griffith and colleagues assessed EMB ocular toxicity in patients receiving mycobacterium avium complex (MAC) treatment [24]. A mean dose of 16.1 mg/kg was administered either daily for three times per week for ± 10.8 months. Patients suffered from reversible ocular toxicity where baseline ocular went back to normal status after discontinuation of EMB. The researchers concluded that three times per week EMB administration was associated with less ocular toxicity than daily EMB administration.

When a subgroup analysis was conducted, hepatotoxicity and gastrointestinal adverse effects incidence was higher in diabetic patients when compared to non-diabetics. This finding is in line with Wu and colleagues where a high number of diabetic patients and an increase in bilirubin was noted [20]. However, they did not correlate the incidence of increase in bilirubin with the existence of diabetes as a chronic disease among the participants. On the other hand, data regarding diabetes effect on efficacy of TB treatment are limited. In one study, diabetic patients had more positive sputum microscopy after 2 months compared to non-diabetics (18% vs. 10%, adjusted OR 1.90; 95% CI, [0.82–4.42]), and 22.2% of diabetic patients had positive sputum culture after 6 months compared to 9.6% who were non-diabetics (adjusted OR, 7.65; 95% CI, [1.89–30.95]) [25].

In the current study, patients with diabetes needed longer time for sputum conversion, which is consistent with previous studies [26–28]. Previous studies conducted on diabetic patients with TB infection showed a slow response to the treatment and lower levels of anti-TB medications compared to non-diabetics [29–31]. However, Ruslami et al. conducted a pharmacokinetic study on 36 patients, 18 of them were diabetic, and concluded that diabetes does not alter pharmacokinetics of anti-TB medications during the intensive treatment phase [32].

There were certain limitations for this study. This was performed retrospectively where the number of patients was relatively small. Hence, a prospective randomized study or a retrospective study with larger number of patients and a close follow-up is warranted in order to confirm the current findings. Another limitation is that the majority of the included patients were Asian males. This may not reflect the general population with pulmonary TB; however, it reflects the infected patients in Qatar who are young foreign workers. In similar studies, the ratio of male to female was smaller than the current study [19, 20]. Furthermore, the study was not powered to detect the difference between diabetic and non-diabetic patients in terms of efficacy and safety. Those finding were found part of a subgroup analysis. Since it is a retrospective study, another limitation is the lack of AFB data at 2, 4 and 6 months that the study group could not obtain.

## Conclusion

Pulmonary TB patients treated with a 4-FDC using Rifafour® showed to have similar response to treatment to those treated with ST while maintaining a similar safety profile, except for visual adverse events that were more noticed in the ST than FDC. Diabetic patients treated with FDC had a faster AFB conversion than those treated with ST regimen. Also, more hepatotoxicity and gastric adverse events were seen among patients with diabetes. Since the current study was not powered to compare the efficacy and safety of FDC to ST, further investigation in this population is warranted.

## Abbreviations

BMI: Body mass index
DOT: Direct observed therapy
EMB: Ethambutol
FDC: Fixed-dose combination
IUATLD: International Union Against Tuberculosis and Lung Disease
INH: Isoniazid
PTB: Pulmonary tuberculosis
PZA: Pyrazinamide
RIF: Rifampin
ST: Separate tablets
SD: Standard deviation
TB: Tuberculosis
WHO: World health organization
